# Characteristics and Developments in Mesenchymal Stem Cell Therapy for COVID-19: An Update

**DOI:** 10.1155/2021/5593584

**Published:** 2021-06-02

**Authors:** Lu Sang, Xiaoqin Guo, Jie Shi, Shike Hou, Haojun Fan, Qi Lv

**Affiliations:** ^1^Institute of Disaster Medicine, Tianjin University, Tianjin, China; ^2^Tianjin Key Laboratory of Disaster Medicine Technology, Tianjin, China

## Abstract

The outbreak of coronavirus disease 2019 (COVID-19) has so far resulted in over a hundred million people being infected. COVID-19 poses a threat to human health around the world. Severe acute respiratory syndrome coronavirus 2 (SARS-CoV-2) has been confirmed as the pathogenic virus of COVID-19. SARS-CoV-2 belongs to the *β*-coronavirus family of viruses and is mainly transmitted through the respiratory tract. It has been proven that SARS-CoV-2 mainly targets angiotensin-converting enzyme II (ACE2) receptors on the surface of various cells in humans. The main clinical symptoms of COVID-19 include fever, cough, and severe acute respiratory distress syndrome (ARDS). Current evidence suggests that the damage caused by the virus may be closely related to the induction of cytokine storms in COVID-19. No specific drugs or measures have yet to be shown to cure COVID-19 completely. Cell-based approaches, primarily mesenchymal stem cells (MSCs), have been identified to have anti-inflammatory and immune functions in COVID-19. Clinical studies about using MSCs and its derivatives—exosomes for COVID-19 treatment—are under investigation. Here, we review the current progress of the biological characteristics, clinical manifestations, and cell-based treatment development for COVID-19. Providing up-to-date information on COVID-19 and potential MSC therapies will help highlight routes to prevent and treat the disease.

## 1. Introduction

Coronavirus is named after the spinous proteins resembling coronae on the surface of the viral envelope. Coronaviruses are widely found in nature. They are respiratory viruses with a range of hosts, including humans, vertebrates, and invertebrates. The occurrence of coronavirus in recent years indicates that it has become one of the major threats to human health [1–6]. The structural characteristics of the coronaviruses and their numerous hosts make them more susceptible to mutations. This brings great difficulty in developing effective preventative and therapeutic options for coronaviruses and their associated diseases [7, 8]. Since December 2019, several cases of viral pneumonia were detected in Wuhan, China. The pneumonia was confirmed to be caused by a novel coronavirus. The first 41 confirmed cases of COVID-19 developed symptoms in December 2019 [8]. According to the World Health Organization (WHO), more than a hundred million cases of COVID-19 patients have been confirmed worldwide [9], including numerous infected health workers. COVID-19 is highly contagious, is susceptible to dyspnea, and presents difficulties when dealing with complex symptoms and complications [10]. In this review, we focus on the biological and clinical characteristics, pathogenic mechanisms, and the current status of treatment, with special focus on MSCs and MSC-derived exosome treatments for COVID-19. This up-to-date account may help to contribute to the further understanding of COVID-19 and to finally achieve improved treatment.

## 2. SARS-COV-2

### 2.1. Discovery of SARS-CoV-2

On the 12th of January 2020, the WHO temporarily named the coronavirus novel coronavirus 2019, or “2019-nCoV,” and the National Health Commission of the People's Republic of China named the disease novel coronavirus pneumonia or “NCP” on the same day. On the 11th of February 2020, the WHO formally named the disease as coronavirus disease 2019 (COVID-19). Meanwhile, the International Committee on Viral Classification formally named the coronavirus causing the disease, as coronavirus type 2 of severe acute respiratory syndrome (SARS-CoV-2) [11].

**Figure 1:**
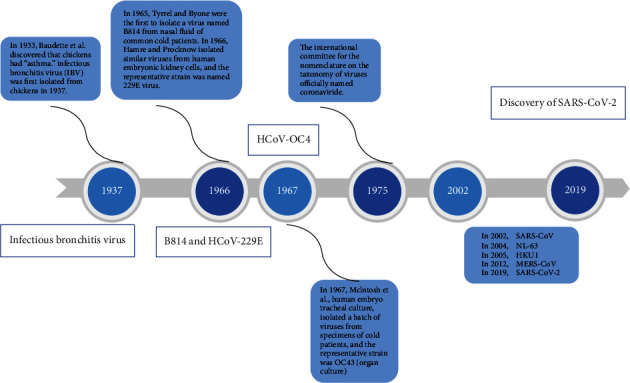
The discovery process of human coronaviruses.

Coronaviruses are single-stranded RNA viruses with a capsule (25-31 kb) [12, 13]. They can cause respiratory, intestinal, liver, and central nervous system diseases [14]. Approximately 20% of common cold infections in humans (less common in children) are caused by coronavirus [15–17]. Since 1965, a total of 7 human coronaviruses have been identified to cause human disease. The discovered human coronaviruses include 229E, OC43, NL63, HKU1, SARS-CoV, Middle East respiratory syndrome coronavirus (MERS-CoV), and SARS-CoV-2 [14, 18, 19]. The recently discovered SARS-CoV-2 is different from SARS-CoV and is considered a novel coronavirus with prominent capacity for human infection [11]. The study on the human routes of infection and transmission of SARS-CoV-2 is important for the effective prevention and treatment of COVID-19. The discovery process of human coronaviruses is shown in Figure 1 [20–22].

### 2.2. Pathogenic Characteristics of SARS-CoV-2

The coronavirus family includes the *α*, *β*, *γ*, and *δ* subfamilies. The *α* and *β* subfamilies mainly infect mammals, whereas the *γ* and *δ* subfamilies are infectious in birds and fish [23]. The coronaviruses, including SARS-CoV-2, SARS-CoV, and MERS-CoV, are *β* species [11, 24, 25]. Negative staining electron micrographs showed that the SARS-CoV-2 particle was generally a pleomorphic sphere with a diameter of about 60–140 nm [22]. Viruses have very distinctive protein spikes of about 9 to 12 nm that make the outer surface of the viruses look like coronae. The key spike (S) proteins bind to human ACE2 receptors and use them as entry point to infect host cells [25]. Studies showed that the similarity of the genome sequences between SARS-CoV-2 and SARS-CoV was approximately 80% [11, 21, 22]. The similarity between SARS-CoV-2 and SARS-like coronaviruses collected from bats in China (bat-SL-CoVZC45 and bat-SL-CoVZXC21) was shown to be 88% [11]. However, later sequencing of the novel coronavirus found in the United States and Italy was not consistent with the earlier sequencing of the novel coronavirus found in China. Therefore, it was proposed that it was highly likely that the novel coronavirus had mutated before February 2020 [26].

### 2.3. Source of Infection

Whether animals are the source of infection has been doubted since the outbreak of a novel coronavirus. SARS-CoV was transmissible through civets, while MERS-CoV was transmissible through camels [27–29]. Studies indicated that SARS-CoV-2 may also be derived from wild animals. Nevertheless, the source of infection was determined to be the seafood market in Wuhan. The natural hosts of SARS-CoV-2 are believed to be bats and/or turtles, snakes, and pangolins, but the exact source of COVID-19 lacks ample evidence [30].

The identification of “patient zero” can help to determine the source of infection, the mode of transmission, whether he/she came into contact with animals, and how he/she came into contact with those animals. The current infection source is mainly human patients that have been infected by SARS-CoV-2. Asymptomatic infected persons may also become infection sources, as the virus has contagious effects during the incubation period [31, 32]. COVID-19 patients discharged from the hospital might show positive nucleic acid testing in the reexamination, whether these patients are still infectious remains to be determined [33]. It is very likely that the disease will persist within the global population, much like the common cold. Furthermore, since SARS-CoV-2 belongs to the plus-strand RNA virus family, it is unstable and likely to mutate, which makes it extremely difficult to prevent and control spreading.

### 2.4. Routes of Transmission

SARS-CoV-2 has a definite human-to-human transmission ability and is mainly transmitted by respiratory droplets and close contact [24, 25, 34–40]. Exposure to high-concentration aerosol for a long time, which is called aerosol transmission, may encourage spread in relatively closed environments [41]. As feces and urine in SARS-CoV-2 nucleic acid tests were shown to be positive for the viral RNA, it is likely to indicate the presence of live viruses in human waste, thus environmental pollution may also be a source of infection [42]. SARS-CoV-2 has the ability to replicate in conjunctival tissues. Indeed, COVID-19 was shown to cause infection through the conjunctiva and also the excreta [43, 44]. Recently, cases in China identified a mutated strain, D614G, which promotes the infectivity of SARS-CoV-2 and enhances viral transmission [45]. Effective methods to eliminate infection sources and transmission of the virus to protect vulnerable groups require urgent address.

## 3. Clinical Characteristics of COVID-19

The general population is susceptible to SARS-CoV-2, but the infection rate in children is relatively low. It remains to be further studied whether this relates to the higher proportion of lymphocytes in children [35, 46]. Respiratory failure is the main reason for aggravation of SARS-CoV-2 and SARS-CoV infections. Overall, the incidence rate is higher in men than in women and the case fatality rate is more than three times higher in women [35, 47]. Women may have a less susceptible advantage.

The incubation period for COVID-19 is generally three to seven days but, maximumly, may take up to fourteen days [48]. It has been reported that the median time from the first symptom to dyspnea is about five days and to ARDS is eight days [34]. Most patients have symptoms of fever, chills, fatigue, dry cough, and muscle pain; a few are accompanied by nasal obstruction, running nose, pharynx galgia and diarrhea, which may or may not be accompanied by pneumonia [25]. Severe and critically ill patients present low to moderate fever over the course of the disease, whereas there have been reported cases of no presentation of significant fever [25]. Severe patients usually have dyspnea and/or hypoxemia one week after onset; critically ill patients may rapidly progress to ARDS, septic shock, difficulty to correct metabolic acidosis, bleeding, and coagulation disorders [34]. The data showed that 80% of these patients were mild and 2% to 5% progressed from mild to severe [49–51]. The initial clinical course of the respiratory disease can be complicated by interstitial pneumonia, 10% to 15% of the patients evolving toward ARDS, who then require mechanical ventilation [52].

The target of SARS-CoV-2 is ACE2 receptors and cells rich in this receptor site are vulnerable to viral attack [30]. The reason that SARS-CoV-2 can spread systemically throughout the blood circulation in the body is that ACE2 are expressed in almost all endothelial cells and smooth muscle cells in organs. All tissues and organs that express ACE2 are susceptible to becoming novel coronavirus and host immune cell battlegrounds. ACE2 receptors are enriched on the surface of the type II alveolar epithelial cells (AT2) and capillary endothelial cells [53]. The intestine is the largest and most complex immune organ in the body; 70% to 80% of the immune cells are in the lymphatic tissues of the intestine. More importantly, ACE2 enzyme presents in abundance on enterocytes of the small intestine and some patients first present with gastrointestinal discomfort [53, 54]. COVID-19 also attacks the heart, lungs, kidneys, and testicles [55–58]. Additionally, facial pain and nasal obstruction are the most common symptoms and the effects related to smell and taste disorders are significantly greater in women than men [59]. Moreover, the disease is easily dangerous, progresses quickly, and can cause multiple organ failure—especially for those patients with preexisting diseases (e.g., diabetes, hypertensive, coronary heart disease, and kidney disease) [60–66]. The prognosis is good in most patients, with relatively mild symptoms in children and few patients progressing to a critical condition [46]. Of all patients, the elderly parts have a particularly poor prognosis [60, 61].

## 4. COVID-19 Pathogenic Mechanisms

Studies have found that SARS-CoV-2, like SARS-CoV, enters into host cells through the combination of the viral spike (S) proteins with the host cell receptor ACE2 [11, 67, 68]. The virus then replicates and spreads in large numbers to trigger an immune response, attracting a large numbers of white blood cells and antibodies to clear the virus. Taken together, SARS-CoV-2-infected patients showed circulating elevated levels of proinflammatory cytokines (such as interferon-*γ* (IFN-*γ*), interleukin- (IL-) 1*β*, IL-6, and IL-12) and chemokines (CXCL10 and CCL2), which are associated with pulmonary inflammation and extensive lung involvement [55]. COVID-19 was characterized by a diminished innate immune response, with reduced expression of genes involved in Toll-like receptors (TLRs) and interleukin signaling, chemokine binding, neutrophil degranulation, and interactions with lymphoid cells [69]. The affinity between SARS-CoV-2 S proteins and the host cell ACE2 is 10- to 20-fold greater than that of SARS-CoV, which may be the primary reason for its greater infectivity [70, 71]. Increased cohesiveness between SARS-CoV-2 S proteins and the host cell ACE2 is evident, with the binding force of SARS-CoV-2 being shown to be approximately 10–20 times higher than that of SARS-CoV, hence the greater intensity of infectivity. As shown by previous data in the literature, the receptor ACE2 is the medium for SARS-CoV-2 to enter the host cells and the serine protease TMPRSS2 is responsible for S protein priming. The S proteins present on the capsid of the virus and bind to the ACE2 receptor on the cells following priming [72]. However, the molecular mechanisms by which SARS-CoV-2 causes the disease and how host-pathogen interactions and host immune responses occur are still relatively unknown.

Most studies speculate that the “cytokine storm” is the cause of severe disease and death. SARS-CoV-2 causes an excessive immune response in the body and induces an inflammatory storm, almost immediately after infection [73]. In the first confirmed patients with severe COVID-19, a large proportion of patients developed cytokine storm syndrome (CSS). That means many immune-active molecules can cause cytokine storm, and subsequently causes ARDS [10, 55, 74]. The representative components involved in cytokine storm development mainly include TNF-*α*, interferons, interleukins, colony-stimulating factors, and chemokines.

Expressions of inflammatory factors in mild and severe COVID-19 patients are different [54, 55, 75, 76]. The pathological results of COVID-19 patients showed that SARS-CoV-2 mainly attacked the lungs, presenting diffuse alveolar injury and lung hyaline membrane formation, which was consistent with ARDS [77]. Monocytes, especially lymphocytes, were observable in lung tissues. The general pathological features were similar to those of SARS and MERS, but the degree of fibrosis is less severe, and the inflammation is more intense. Furthermore, CD4+ and CD8+ T lymphocytes in peripheral blood were significantly reduced. However, the increased proportions of highly stimulated CCR4+CCR6+ Th17 subset cells and CD8+ T cells were found to contain high concentrations of cytotoxic particles; both revealed dysregulation of the T lymphocytes and hyperactivated status of lymphocytes [77]. This implied that the abnormal activation and imbalance of T lymphocyte subsets were the key to the process of CSS of COVID-19. Patients who have hyperinflammation suffered more risk of mortality [74]. These abnormal immune responses can lead to long-term lung damage and fibrosis in intensive care survivors and may lead to dysfunction and reduced quality of life. Data was reported that showed that 26% COVID-19 patients developed ARDS and more than 90% of nonsurvivors had developed ARDS. Furthermore, a proportion of patients may develop irreversible pulmonary fibrosis [78]. Therefore, it is important to treat this condition early and aggressively to prevent more severe infections that require therapeutic interventions, which can inhibit excessive inflammation and prevent organ damage and long-term dysfunction in severe disease cases.

## 5. Current Therapy for COVID-19

In general, COVID-19 is an acute self-limiting disease [79, 80]. A proportion of patients can be asymptomatic or self-healing after being infected with the virus and can show no symptoms of discomfort. The main clinical treatment for COVID-19 is antiviral therapy supplemented by supportive therapies such as oxygen therapy and mechanical ventilation. Once severe pneumonia or respiratory failure occurs, these comprehensive supportive therapies must be taken into account. There are no available specific treatments for reducing mortality or morbidity until now. In addition, previous studies have reported that convalescent plasma therapy has also shown some therapeutic effect and therefore mainly can help COVID-19 and respiratory failure patients to stop viral shedding and help prolong survival but does not reduce mortality in patients with severe end-stage disease [81]. Lung transplantation is considered when traditional medicine therapy, mechanical ventilation, and extracorporeal membrane oxygenation (ECOM) cannot improve lung function [82]. Lung transplantation is not a routine treatment for COVID-19, but it is currently the only effective clinical treatment for end-stage pulmonary disease. Moreover, abnormalities in host immunity and inflammatory response are the underlying pathogenesis of SARS-CoV-2 and are associated with high mortality from COVID-19. Therefore, treating severe cases of COVID-19 requires not only suppressing virus replication but also preventing and reversing cytokine storms. Researchers found that remdesivir [83, 84], darunavir, and arbidol could inhibit SARS-CoV-2 virus in vitro [85]. Zhao et al. found that glucocorticoid can significantly reduce the toxic symptoms of pulmonary infection in severe SARS patients but it can also reduce the ability to fight infection and cause serious sequelae, such as osteonecrosis of the femoral head [86, 87]. This result suggested that glucocorticoids should be used with careful consideration. China's experience advocates that the treatment must be integrated with traditional Chinese medicine and Western medicine, giving full play to the advantages of Chinese medicine, for example, Lian-Hua Qing-Wen [88]. It is worth noting that the use of renin-angiotensin-aldosterone system (RAS) inhibitors could increase ACE2 levels, which may in turn increase the risk of COVID-19 infection. Gurwitz proposed that inhibitors of RAS, especially angiotensin receptor 1 (AT1R) blockers, may play a therapeutic role in host responses to the virus in COVID-19 patients [89]. A clinical trial (NCT04318418) from Italy is aimed at retrospectively examining whether COVID-19 patients receiving or not receiving AC-I or ARB treatment are at higher or lower risk of developing severe COVID-19 [90]. Due to the important role of inhibitors of RAS, particularly ARBs in cardiovascular and renal diseases, attention should be paid to whether patients with preexisting diseases coexisting with COVID-19 should take ACE inhibitors or angiotensin-receptor blockers [91, 92].

China has successfully developed a candidate vaccine of BBIBP-CorV with effective protection against SARS-CoV-2 and recently entered phase III clinical trials [93]. According to data available to the Chinese public, 15 COVID-19 vaccines have entered clinical trials in China, among which 6 have entered phase III clinical trials [94]. Biomedical companies including Pfizer and BioNTech also announced the completion of phase III clinical trials of the BNT162b2 vaccine. Their final data from phase III clinical trials showed that the vaccine demonstrated over 90% effectiveness. On the 11th of December 2020, the US Food and Drug Administration (FDA) approved applications from Pfizer and BioNTech for Emergency Use Authorization (EUA) for COVID-19 vaccines. The rapidly advancing mRNA vaccine, mRNA-1273 from Moderna in the United States, ended phase III clinical trials and has since entered clinical use. The vaccine was declared to have an effectiveness of 94.5% during phase III clinical trials and was effective in reducing the likelihood of infection progressing to severe disease. The Moderna vaccine will be the second vaccine in the United States made available to help prevent COVID-19. Moreover, the Moderna vaccine induced an immune response in the elderly that was similar to that seen in younger participants, providing a new hope that the vaccine will be effective for those considered to be at high risk of severe illness due to novel coronavirus transmission.

Severe and critical cases lack specific treatments. The current principle of treatment is to treat complications of the disease, prevent secondary infection, and provide functional support to at-risk organs. Blood purification and artificial ECMO technological advancements have led to their use as lifesaving treatments for ARDS and refractory respiratory failure. Nevertheless, the potential compound immune damage associated with an extracorporeal circuit initiation during ECMO needs to be considered [95].

## 6. MSCs: A Promising Treatment

Researchers are now turning focus to the development of pathogen-specific drugs to address host immune and inflammatory abnormalities with cell therapy, to curb high mortality rates for SARS-CoV-2 and COVID-19. Host-directed therapy (HDT) advancements have provided options to both modulate the immune response and inhibit excessive inflammation. Stem cell therapy is a promising treatment that has been used as a treatment for a variety of refractory diseases involving diabetes, bone disease, and neurological diseases, and it has been used to repair and regenerate damaged or lost tissues [96–98].

MSCs are important members of the stem cell family and are derived from the mesoderm and ectoderm in early development. MSCs are pluripotent stem cells, first discovered in bone marrow in 1968 [24, 99]. They are one of the most widely used stem cells and have the advantage of an extensive source. MSCs are mainly found in the connective tissue and interstitium of organs of the body [100, 101], and they come from a variety of sources, including the umbilical cord, bone marrow, adipose tissue, and endothelial progenitor cells [99, 102]. MSCs have high amplification ability, and the gene stability remains viable after multiple passages in vitro. Moreover, MSCs have multidifferentiation potential and can repair various tissues and organs. Additionally, they can be administered to the body through various ways with low immunogenicity. The expression of the class II major histocompatibility complex (MHC-II) and costimulatory molecules is low on the MSC surface [103]. MSCs display remarkably high resistance to infection by viruses [104]. Specifically, IFN is produced only when a virus invades, which activates virus-resistant genes and recruits immune cells to fight viral infections. Pluripotent cells can continuously activate many antiviral genes without relying on IFN [104, 105]. Due to the properties of immune regulation, paracrine, directional chemotaxis of tissue damage, and the advantages of no ethical issues [106–108], MSCs can serve as a promising tool for treating COVID-19 patients.

### 6.1. Therapeutic Effects of MSCs on COVID-19

In many acute lung injury (ALI) and ARDS mouse models, MSC treatment can reduce lung inflammatory injury and edema, improve oxygenation, prevent the development of ARDS, and significantly prolong the survival of mice. [109] Studies have found that MSCs significantly reduced ALI associated with the influenza H5N1 virus and prolonged survival [109]. Notably, the spectrum of inflammatory cytokines induced by H5N1 and COVID-19 is similar [110]; extremely high concentrations of IL-6, GCSF, IP10, MCP-1, MIP1A, and TNF-*α* and may lead to a large degree of severe organ damage and subsequent death [34, 55]. Recent studies have shown that MSCs that secreted exosomes effectively reduced the level of detectable fibrosis through reduced collagen deposition and restored alveolar epithelial structure in bleomycin-induced pulmonary fibrosis in mice and restored normal lung structure [111].

Bone marrow MSCs are widely used in cell therapy, including in a large number of preclinical studies and basic trials [112, 113]. Many clinical trials have demonstrated the safety and efficacy of MSCs. Recent studies reported on multiple clinical trials that used stem cell therapy for COVID-19. A 70-year-old critically severe female COVID-19 patient from Kunming, China, was reported to have a significant improvement in lung function and reduction in clinical symptoms after bone marrow MSC infusion [114]. More than sixty clinical trials have commenced to investigate the use of MSCs for COVID-19 treatment. Seven clinical trials, including from the United States, China, Pakistan, Turkey, Japan, and Indonesia, have recently concluded and are marked “completed” on clinicaltrials.gov. [115] (Table 1). The registered trials have different designs, especially with dose administration and primary schedules, which suggest that there is a lack of global consensus for MSC therapy. Wang and his team successfully completed phase II clinical trials, which included one hundred patients [116]. Notably, they showed no serious infusion-associated adverse events that were observable during UC-MSC intravenous infusion [117]. Outcomes of this clinical trial showed that stem cells inhibited lung inflammation, attenuated abnormal immune activation, and reduced lung injury. Stem cell therapy accelerated the shortening of the course of the disease, and severe patients did not progress to the critical stage. A study by Leng et al. demonstrated that MSC transplantation was safe and effective in patients with COVID-19 with pneumonia (two mild, four severe, and one critical), especially for critically ill patients [49].

### 6.2. Potential Mechanisms of MSCs in Treatment of COVID-19

The underlying mechanism of improvement after infusion of MSCs in COVID-19 patients also showed the strong anti-inflammatory activity of MSCs. It is clear that the results showed effectiveness, including increased peripheral blood lymphocyte count, reduction of C-reactive protein, and immune cell (CXCR3 + NK cells, CXCR3 + CD4 + T cells, and CXCR3 + CD8 + T cells) secretion of overactive cytokines into the blood [49]. Meanwhile, in the MSCs group, IL-10 levels were higher and TNF-*α* were significantly lower [49]. In addition, the gene expression profile showed that MSCs were ACE2 and TMPRSS2, indicating that MSCs had no risk of COVID-19 infection and further characterizing MSC expression profiles [49]. Notably, studies also showed that after SARS-CoV-2 infection, even in older patients, MSCs given intravenously were capable of regulating the pulmonary microenvironment and had immunomodulatory activity, thereby promoting tissue repair while dampening the immune system [49]. Intravenous injection of bone marrow MSCs usually causes them to accumulate in the lungs and increased secreted paracrine factors within the lung tissue [118]. The secreted paracrine factors contributed significantly to the regeneration of alveolar epithelial cells, the resolution of fibrosis, and the restoration of lung function [65].

### 6.3. Immunomodulatory Functions of MSCs

Abnormal activation of innate immunity and adaptive immunity was found in human COVID-19, wherein uncontrolled inflammatory responses led to local and systemic tissue damage. In the human body, ACE2 is expressed by both monocytes and macrophages. Therefore, these cells can be infected by SARS-CoV and SARS-CoV-2 [119], leading to the activation and transcription of proinflammatory genes [120]. Patients with COVID-19 exhibited activated phenotypic morphology (FSC-high) and production capacity for IL-6, IL-10, and TNF-*α*, despite normal monocyte blood counts [121]. Moreover, in bronchoalveolar lavage fluid, proinflammatory macrophages were higher in severe COVID-19 patients, compared to mild patients, which contributed to a cytokine storm [122]. Elevated levels of neutrophils or macrophages may help predict disease severity and clinical outcome [123]. Meanwhile, COVID-19 can also affect the adaptive immune system. Reduction in lymphocytes is a common feature, especially in those patients with severe and critically ill COVID-19. Clinical studies have shown that a decrease in the frequency of circulating lymphocytes, including CD4+ and CD8+ T cells, is strongly associated with the severity of COVID-19 [124]. The negative relationship between proinflammatory cytokines and circulating T cells is likely related to the redistribution of cells in the tissue and/or induction of cell apoptosis [125]. However, activated Th1 cells, together with Th17 cells could directly stimulate monocytes to secret proinflammatory cytokines and accelerate cytokine storm [119, 126]. Therefore, impaired and unbalanced innate and adaptive immune responses are involved in the process of COVID-19.

MSCs have the potential to exert immunomodulatory functions in both innate and adaptive immune responses, including direct and indirect interactions with immune cells [106]. Early studies have demonstrated that MSCs can promote the polarization of monocytes/macrophages toward an anti-inflammatory type 2 subtype, which is characterized by high levels of IL-10 and decreased IL-12 and TNF-*α* production [127, 128]. Moreover, MSCs affected the subsequent activation of antigen-specific CD4+ T cells by inhibiting the expression of MHC class II and CD86 on macrophages. MSC-derived paracrine factors, for example, prostaglandin E2 (PGE2), may be associated with anti-inflammatory and immunosuppressive functions of MSCs [129, 130]. In addition, transforming growth factor-*β* (TGF-*β*) is also closely related to the immunosuppressive effects of MSCs on natural killer (NK) cell proliferation and proinflammatory cytokines produced by NK cells [131]. T cells are an important part of the adaptive immune system and play a key role in the body's specific immune response. The abnormal activation of T lymphocytes is an important cause of CSS and ARDS in COVID-19. More than 30 soluble factors, such as TGF-*β*, PGE2, indoleamine 2,3-dioxygenase (IDO), and hepatocyte growth factor (HGF) have been demonstrated to exert the immunomodulatory capacity of MSCs by inhibiting CD4+ Th1 and Th17 cells and CD8+ T cell proliferation and inducing Foxp3+ Treg differentiation [132, 133]. In addition, MSCs suppressed IFN-*γ* released by Th1 and increased IL-4 production from Th2 [134]. Therefore, MSCs can reduce the secretion of inflammatory factors involved in CSS and increase the secretion of anti-inflammatory factors by regulating the innate and adaptive immunity, which can help treat and reduce the mortality of COVID-19 patients [49].

### 6.4. Reparative Functions of MSCs

MSC influences over the repair of tissue defects have been extensively reported [135–137]; MSCs are adult stem cells which can keep a proliferating state for long periods. Additionally, they remain undifferentiated and differentiate into a variety of cell types including cardiomyocytes, hepatocytes, neurons, and astrocytes [138–140]. It has been reported that the immunomodulatory properties of MSCs are one of the main factors in the process of lung repair and regeneration in pathologic conditions such as ALI, chronic obstructive pulmonary disease, bronchopulmonary dysplasia, and idiopathic pulmonary fibrosis [99, 102, 141]. COVID-19 mainly causes lung injury, which is manifested by damage to alveolar epithelial cells and capillary endothelial cells, as well as diffuse pulmonary interstitial and alveolar edema, leading to acute hypoxic respiratory insufficiency and eventually ARDS.

MSCs can affect P13, MAPK, NF-*к*B, and other signaling pathways through potential differentiation ability, the production of cytokines, and the secretion of a large amount of exosomes and vesicles containing microRNA (miRNAs) to repair lung injury [142]. MSCs partially gathered in the injured lung after intravenous injection can differentiate into alveolar epithelial cells and pulmonary vascular endothelial cells, but the efficiency to do so was reported to be extremely low [143]. Moreover, evidence suggests that MSCs express a variety of growth factors and are involved in the regulation of cell proliferation, apoptosis, and differentiation. MSCs can secrete cell nutrition factors (such as keratinocyte growth factor (KGF), HGF, Ang-1, and granulocyte macrophage colony stimulating factor (GM-CSF), while reducing lung tissue expression of TGF-*β*, TNF-*α*, type I collagen, and type III collagen [144, 145]. It has been proven that MSC therapy can secrete extracellular vesicles enriched with HGF, which protected or restored alveolar epithelium and lung endothelial cells and reduced the inflammatory response and increased autophagy [146–149]. MSC therapy have the ability of regeneration through activation of the WNT/catenin signaling pathway, which has also been shown to promote the direct differentiation of MSCs into type II alveolar epithelial cells [150–153]. Moreover, MSCs protected alveolar epithelial cells from inflammatory and oxidative stress damage by secreting IL-1, Ang-1, PGE2, and HGF or by scavenging oxidants and free radicals [154–157]. MSCs were also shown to regulate tissue remodeling processes and attenuated lung fibrosis by increasing metalloproteinase- (MMP-) 8 and decreasing the levels of tissue inhibitors of metalloproteinase- (TIMP-) 1, IL-1*β*, and TGF-*β*1 in animal models of ARDS [158, 159].

Vascular endothelial growth factor (VEGF), epidermal growth factor (EGF), and HGF are factors with important implications in epithelial maturation, regeneration, and repair of the alveolar epithelial barrier during the recovery process of ARDS lung injury [160–162]. Meanwhile, MSCs accumulated in the lungs after intravenous infusion, which improved the tissue microenvironment to one more conducive for resident lung cells, proangiogenic cells, and proregenerative cells [136, 137, 163, 164]. Bone marrow MSCs accelerate healing through regulatory mechanisms that affect immunoregulation, apoptosis, and angiogenesis, while supporting the recruitment, growth, and differentiation of local stem cells and progenitor cells.

As ARDS worsens, multiple organ failure may occur, leading to an increase in morbidity and mortality. MSC administration can reduce the histopathological impairment of lung tissues and promote functional recovery in ARDS models [153, 159, 165]. MSCs also improved the repair and functional recovery in other distal organs, including the heart [166], liver [159, 167], kidney [159, 168], and gut [169, 170]. Therefore, MSC administration can not only improve the recovery of lung function but also delay or inhibit the development of ARDS to multiorgan injury.

## 7. Prospects

**Figure 2:**
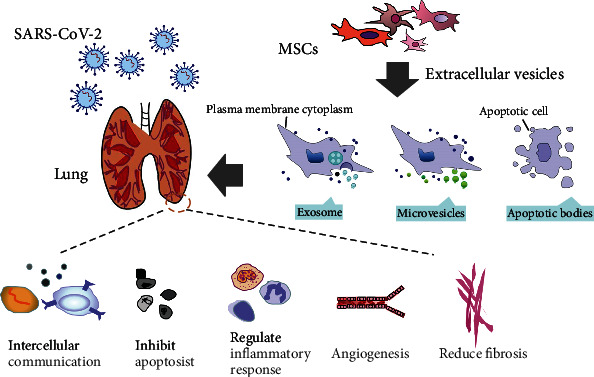
SARS-CoV-2 enters into the human lung and the MSCs-EVs are considered a promising treatment due to their effects.

The anti-inflammatory effects of MSCs were mainly attributed to the release of paracrine factors, despite the poor survivability of donor-derived MSCs in the host lung tissue after intravenous treatment or lung engraftment [163, 171]. Nevertheless, there are many hurdles to overcome before it can be applied to clinical trials, including the heterogeneity of cells, large-scale production, and harsh storage conditions; it also highly possibly causes tumor formation after ectopic engraftment [172]. So its use is restricted by security consideration. Recent studies showed that certain therapeutic effects of MSCs depend on their ability to secrete extracellular vesicles (EVs). Extracellular vesicles are secreted by nearly all mammalian cell types with a diameter of approximately 20–2000 nm [173]. The extracellular vesicles were identified as unneeded compounds of cells initially [174]. To date, we know that extracellular vesicles are responsible for the component exchange and communication between cells instead of just waste carriers and act as signaling vehicles to recipient or as a consequence of pathological developments [175–177]. According to the recent statement from the International Society for Extracellular Vesicles (ISEV) in 2018, EVs are generally classified into exosomes, microvesicles (MVs), and apoptotic bodies (ABs) according to their sizes, surface markers, and cellular generation mechanism [178, 179] (Table 2). EVs can transfer mRNA, microRNA, proteins, lipids, and even organelles such as mitochondria to target cells and tissues, change gene expression, and regulate target cell behavior to reduce the inflammatory response, thus mediating and mimicking therapeutic roles of their parental MSCs (Figure 2) [180–182]. Moreover, there is accumulating evidence indicating that MSC-EVs have shown equal or even better treatment efficacies than MSCs in many diseases. EVs can be considered as potent reservoirs of bioactive substances within the MSC secretome [183, 184].

Extracellular vesicles are released by various cell types, including epithelial cells, tumor cells, macrophages, and MSCs, which are believed to play a unique role in intercellular communication. MSC-EVs are composed of soluble proteins, including a variety of cytokines, chemokines, and growth factors [185]. Once released, EVs and soluble proteins interact with the target cell and regulate the cellular response. MSC-EVs, unlike monoclonal antibodies, can act simultaneously and possibly synergistically on many cytokines. Other than that, the functions of EVs mainly are identified to depend on the cell origin. EVs can activate endogenous stem cells, progenitor cells, and genetic material transfer; inhibit cell apoptosis; regulate inflammatory response; stimulate extracellular matrix remodeling, angiogenesis, and reduce fibrosis; and mediate chemotaxis [186]. Moreover, EVs are generally considered safer than cell therapy because they lack the capacity to induce endogenous tumor formation, while possessing low immunogenicity. In addition, EVs are easier to manipulate and store than cells and, thus, cost less. More importantly, EVs can take advantage of multitargeted therapy, which is far superior to monotherapy, especially in instances of COVID-19 multiorgan dysfunction [187]. Therefore, MSC-EVs are a promising tool for cell-free therapy of pulmonary diseases and may be more suitable for human use as COVID-19 therapy.

Preclinical trials have proven that MSC-derived EVs can be used as acellular substitutes for ARDS therapy [182]. Indeed, MSC-EV infusion can reduce proinflammatory factors and the subsequent cytokine storm that drives ARDS progression. Meanwhile, there is also a corresponding increase of anti-inflammatory signaling mediators, which reduce the severity of lung injury by increasing the permeability and function of the alveolar epithelium [182]. Furthermore, virus reproduction could be inhibited by MSC-derived EVs directly [188, 189]. A key intrinsic component of EVs is miRNA, which is closely associated with physiological processes such as development and immunoregulation through epigenetic alterations [190]. Packaged miRNAs in EVs influence the differentiation and function of multiple types of cells, and excessive levels are associated with a variety of diseases, including cancer, lung diseases, obesity, diabetes, and cardiovascular disease [191–196].

Studies demonstrated that miRNA derived from MSC EVs could alter epigenetic activity, leading to changes in cell receptor expression and the subsequent exchange of genetic material, which also helped prevent RNA viruses, including coronaviruses from cell entry [188]. In a swine influenza model, infection and virus shredding were significantly inhibited following the administration of MSC-derived EVs [197]. Studies have utilized different techniques to track the biological distribution of EVs throughout the body in different animal models. EV tracking allowed researchers to show that in rat models of ALI, intravenously injected EVs reached lung tissues [198]. Numerous studies have shown that EVs from various sources could restore lung injury, improve respiratory function, and, in some cases, improve survival. EV-based therapies for COVID-19-related lung injury have great potential because they target multiple pathways implicated in COVID-19 insult while enhancing tissue regeneration.

As evidenced by an increase in the number of EV studies, recently published, EV therapy remains a popular research field and continues to receive a high amount of attention. The literature reported that the clinical efficacy and safety of MSC products in preclinical models of pulmonary diseases and the indication that noninvasive methods can be utilized (e.g., inhalation) offer novel perspectives [199]. A previous study by Dinh et al. demonstrated that inhalation of globular cell secreted exosomes in mice treated with different lung injury patterns and promoted pulmonary fibrosis repair [111].  Table 3 lists the clinical trials applied for COVID-19: MSC-derived extracellular vesicle treatment for COVID-19 registered on clinicaltrials.gov [200]. To be specific, clinical trials (NCT04276987 and NCT04491240) registered by China and Russia are using aerosol inhalation of MSC-derived exosomes to treat COVID-19 and have been marked as “completed” on clinicaltrials.gov. NCT04491240 has reported some results. The researchers found that the MSC exosome treating reduced the C-reactive protein (CRP) and lactic acid dehydrogenase (LDH) level in serum and no adverse reactions, such as allergy and bronchospasm, were found during the test. A nonrandomized open-label cohort study reported that born marrow MSC-derived exosomes could improve oxygenation, reduce neutrophil count, elevate average CD3+, CD4+, and CD8+ lymphocyte counts, and reduce the levels of acute-phase reactants in severe COVID-19 patients [201]. These findings demonstrated the potential of MSC-derived exosomes for the treatment of COVID-19 patients. However, even though the generic term extracellular vesicles are currently used to refer to these secreted membrane vesicles, they are in fact highly heterogeneous. Before converting EV therapy into human clinical applications, efforts should be made to address many issues such as the optimal dose and route of administration in animal models. Although there are many obstacles and drawbacks that need to be addressed, inhaled drug administration activity in these studies could provide a way to treat lung injury in COVID-19 pneumonia.

MSCs remain the most relevant stem cell technology for a wide range of diseases. On the other hand, delivery via EVs offers an opportunity to avoid the use of cell therapy. Although this requires further research, it may help ensure the safety of MSC treatment. MSCs and their secretome are being used to study a large number of diseases involving skin pathology, cardiovascular disease, neuropathology, metabolic disorders, spinal cord injury, and autoimmune diseases [202]. We are encouraged that other sources of MSCs are gradually expanding including the embryonic stem cells (ESCs) and induced pluripotent stem cell- (iPSC-) derived MSCs, ESC-MSCs, and iPSC-MSCs [202]. These have been shown to overcome age-related problems and limited proliferation rates of adult MSCs. In addition, iPSC-MSCs showed signs of rejuvenation. Although many clinical trials have been conducted and clinical trial details can be found at ClinicalTrials.gov, there are fewer and less consistent clinical trials to complete, so we need to continue to pay attention to this aspect [203].

The current epidemic has entered a severe and complex period. By April 2021, more than a hundred million people worldwide have been diagnosed with COVID-19, with more severe cases being reported daily. For severely ill COVID-19 patients, it is more important to treat CSS, ARDS, and ALI function in addition to routine antiviral therapy, to reduce the mortality rate. We believe that both MSCs and secreted exosomes have great potential in repairing lung injury. Therefore, they have the potential to serve as promising treatment options for severe COVID-19.

## 8. Conclusion

COVID-19 is at a critical stage wherein control is paramount, but there are still no targeted or highly effective treatment options for patients with severe COVID-19 or those that are clear from infection but must live with lung complications and other consequences of the disease. MSC therapy has played an important role in clinical trials. Although MSC therapy has many difficulties to overcome before reaching its full therapeutic potential (source, heterogeneity, and quality control), its derivative EVs have attracted attention and have been tested in COVID-19 clinical trials with promising results. However, current data is limited and a better understanding of stem cell therapy and EV therapy and their safety development into effective preclinical endpoints is needed to recognize and address these therapeutics and their progress toward truly meeting the clinical needs of COVID-19.

## Figures and Tables

**Table 1:** Clinical trials investigated the use of MSCs for COVID-19 treatment marked “completed” on clinicaltrials.gov.

Trial ID no.	Status	Responsible country	Study title	Interventions
NCT04288102	Completed	China	Treatment with human umbilical cord-derived mesenchymal stem cells for severe corona virus disease 2019 (COVID-19)	UC-MSCs
NCT04355728	Completed	United States	Use of UC-MSCs for COVID-19 patients	UC-MSCs
NCT04492501	Completed	Pakistan	Investigational treatments for COVID-19 in tertiary care hospital of Pakistan	MSCs
NCT04522986	Completed	Japan	An exploratory study of ADR-001 in patients with severe pneumonia caused by SARS-CoV-2 infection (COVID-19)	AD-MSCs
NCT04535856	Completed	Indonesia	Therapeutic study to evaluate the safety and efficacy of DW-MSC in COVID-19 patients (DW-MSC)	MSCs
NCT04573270	Completed	United States	Mesenchymal stem cells for the treatment of COVID-19	UC-MSCs
NCT04713878	Completed	Turkey	Mesenchymal stem cells therapy in patients with COVID-19 pneumonia	MSCs

UC-MSCs: human umbilical cord mesenchymal stem cells; AD-MSCs: adipose-derived mesenchymal stem cells.

**Table 2:** The characteristics and categories of EVs.

Characteristic	Exosomes	Microvesicles	Apoptotic bodies
Apoptotic bodies	50–150	100–1000	500–2000
Origin	Multivesicular body exocytosis	Cell membrane budding and fission	Plasma membrane, endoplasmic reticulum
Morphology	Cup/round shaped	Various shapes	Heterogeneous
Sucrose gradient	1.13–1.19 g/mL	1.04–1.07 g/mL	1.16–1.28 g/mL
Surface markers	Annexins, tetraspanins, heat-shock proteins	CD40, cholesterol, sphingomyelin, ceramide	Annexin V positivity, TSP, C3b
Contents	Proteins, nucleic acids, lipid	Proteins, nucleic acids, lipid	Nuclear fractions, DNA, cell organelles
Isolation technique	Centrifugation at 100,000 gravity	Ultracentrifugation	Ultracentrifugation

**Table 3:** MSC-EV treatment trials in COVID-19 registered on Clinical Trials. The treatment of COVID-19 disease, including trial numbers, source of EVs, routes of administration, and primary endpoints.

Trial ID no.	Status	Responsible country	Source of extracellular vesicles	Administration	Primary endpoints
NCT04276987	Completed	China	Exosome derived from allogenic adipose mesenchymal stem cells	Inhalation	Adverse reaction
NCT04491240	Completed has results	Russian Federation	MSC exosomes	Inhalation	Adverse events
NCT04493242	Not recruiting	United States	Bone marrow-derived extracellular vesicles	IV	All-cause mortality
NCT04602442	Enrolling by invitation	Russian Federation	Mesenchymal stem cell exosomes	Inhalation	Adverse events during trial
NCT04657458	Available	United States	Bone marrow-derived extracellular vesicles	IV	Not recorded
NCT04798716	Not recruiting	United States	MSC exosomes	IV	Adverse events

## Data Availability

The processed data are available from the corresponding author upon request.
